# Genomic evidence for genes encoding leucine-rich repeat receptors linked to resistance against the eukaryotic extra- and intracellular *Brassica napus* pathogens *Leptosphaeria maculans* and *Plasmodiophora brassicae*

**DOI:** 10.1371/journal.pone.0198201

**Published:** 2018-06-01

**Authors:** Henrik U. Stotz, Pascoe J. Harvey, Parham Haddadi, Alla Mashanova, Andreas Kukol, Nicholas J. Larkan, M. Hossein Borhan, Bruce D. L. Fitt

**Affiliations:** 1 School of Life and Medical Sciences, University of Hertfordshire, Hatfield, United Kingdom; 2 Saskatoon Research Centre, Agriculture and Agri-Food Canada, Saskatoon, SK, Canada; 3 Armatus Genetics Inc., Saskatoon, SK, Canada; New South Wales Department of Primary Industries, AUSTRALIA

## Abstract

Genes coding for nucleotide-binding leucine-rich repeat (LRR) receptors (NLRs) control resistance against intracellular (cell-penetrating) pathogens. However, evidence for a role of genes coding for proteins with LRR domains in resistance against extracellular (apoplastic) fungal pathogens is limited. Here, the distribution of genes coding for proteins with eLRR domains but lacking kinase domains was determined for the *Brassica napus* genome. Predictions of signal peptide and transmembrane regions divided these genes into 184 coding for receptor-like proteins (RLPs) and 121 coding for secreted proteins (SPs). Together with previously annotated NLRs, a total of 720 LRR genes were found. *Leptosphaeria maculans*-induced expression during a compatible interaction with cultivar Topas differed between RLP, SP and NLR gene families; NLR genes were induced relatively late, during the necrotrophic phase of pathogen colonization. Seven RLP, one SP and two NLR genes were found in *Rlm1* and *Rlm3*/*Rlm4*/*Rlm7*/*Rlm9* loci for resistance against *L*. *maculans* on chromosome A07 of *B*. *napus*. One NLR gene at the *Rlm9* locus was positively selected, as was the RLP gene on chromosome A10 with *LepR3* and *Rlm2* alleles conferring resistance against *L*. *maculans* races with corresponding effectors *AvrLm1* and *AvrLm2*, respectively. Known loci for resistance against *L*. *maculans* (extracellular hemi-biotrophic fungus), *Sclerotinia sclerotiorum* (necrotrophic fungus) and *Plasmodiophora brassicae* (intracellular, obligate biotrophic protist) were examined for presence of RLPs, SPs and NLRs in these regions. Whereas loci for resistance against *P*. *brassicae* were enriched for NLRs, no such signature was observed for the other pathogens. These findings demonstrate involvement of (i) NLR genes in resistance against the intracellular pathogen *P*. *brassicae* and a putative NLR gene in *Rlm9*-mediated resistance against the extracellular pathogen *L*. *maculans*.

## Introduction

Eukaryotic plant pathogens have developed different strategies of host colonization to acquire nutrients and reproduce. Obligate biotrophic pathogens directly penetrate and manipulate living host cells for nutrition, growth and sporulation. In contrast, necrotrophic pathogens rapidly kill host tissues to feed on dying plant cells. Furthermore, extracellular hemibiotrophic fungal pathogens colonize the apoplast and grow like endophytes for a long phase of their life cycles, then switch to a necrotrophic phase of growth [1]. Recognition of pathogens by host plants differs, depending on their attack strategies. Intracellular nucleotide-binding leucine-rich repeat (LRR) receptors (NLRs) of plants recognize effectors, produced by cell-penetrating intracellular eukaryotic biotrophic pathogens and targeted into the cytoplasm of host cells, or their cytoplasmic targets. In contrast, host membrane-bound receptor-like proteins (RLPs) that contain extracellular LRR (eLRR) domains can recognize effectors of extracellular fungal pathogens. Examples include tomato *Cf* and apple *HcrVf2* genes that confer resistance against *Cladosporium fulvum* and *Venturia inaequalis*, respectively [1–3]. RLPs, encoded by *Ve* genes, also operate in race-specific resistance against *Verticillium albo-altum* [4]. A need for analyzing eLRR receptor genes was recently stated [5]. Although the focus of this study was on other LRR genes, we acknowledge that some receptor-like kinases (RLKs) are involved in *R* gene-mediated resistance [6–8].

Recognition of necrotrophic pathogens may be less specific but may depend on host perception of pathogen- or damage-associated molecular patterns. Genetic evidence suggests that the *Arabidopsis thaliana* gene *AtRLP30* is involved in perception of a protein secreted by *Sclerotinia sclerotiorum* [7]. Whereas *Atrlp30* mutants are more susceptible to two necrotrophic pathogens and the non-pathogenic bacterium *Pseudomonas syringae* pv. *phaseolicola* [7, 9], resistance against the extracellular hemibiotrophic fungal pathogen *Leptosphaeria maculans* remains unaltered [10]. Besides NLRs and RLPs, a class of secreted proteins (SPs) exists that merely contain a LRR domain [11]. Some host SPs interact with microbial polygalacturonases to inhibit degradation of plant cell walls by pathogenic fungi [12].

Examples of intracellular obligate biotrophic, extracellular hemi-biotrophic and necrotrophic eukaryotic pathogens of oilseed rape (*Brassica napus*) that cause significant yield losses on different continents include *Plasmodiophora brassicae* (clubroot), *L*. *maculans* (phoma stem canker) and *S*. *sclerotiorum* (stem rot), respectively. Monogenic resistance (*R*) genes operate against *P*. *brassicae* and *L*. *maculans* but probably not against *S*. *sclerotiorum* [13–16]. The *Crr1a* gene, which confers resistance against *P*. *brassicae* in *Brassica rapa*, is located on chromosome A08 and codes for an NLR with an N-terminal Toll-Interleukin-1 receptor (TIR) domain [15]. The *Rcr1* locus for resistance against *P*. *brassicae* comprises five genes, four of which encode TIR-NLRs [17]. The *P*. *brassicae* genome shares many features with other obligate biotrophic pathogens and also may be representative of filamentous fungi and oomycetes [18]. A cluster of four *R* genes (*Rlm3*, *Rlm4*, *Rlm7* and *Rlm9*) and the *Rlm1* locus for resistance against *L*. *maculans* are located on *B*. *napus* chromosome A07 [19, 20]. Two alleles, *LepR3* and *Rlm2*, of the same *R* gene on chromosome A10 encode an RLP that confers resistance against *L*. *maculans* races with *AvrLm1* and *AvrLm2* genes that express the corresponding effectors, respectively [13, 14].

Here, the hypothesis that eLRR and NLR genes control resistance against extracellular (apoplastic) and intracellular (cell-penetrating) eukaryotic pathogens, respectively, was examined. Genome sequences of *B*. *napus* cv. Darmor-*bzh* [21] and DH12075 [4] were used to determine the distribution, expression [22] and evolution of candidate resistance genes. Locations and physical distances of mapped resistance loci were used to identify putative *R* genes against *L*. *maculans* on chromosome A07. These candidate genes were examined, based on the assumption that *R* genes are under positive selection [23, 24]. Physical mapping data for resistance against *P*. *brassicae*, *L*. *maculans* and *S*. *sclerotiorum* were used to examine the importance of RLPs, SPs and NLRs in resistance against eukaryotic pathogens with three different colonization strategies. This study contributes to a genome-wide understanding of the function of distinct LRR receptors in resistance against three pathogens of oilseed rape.

## Results

### Distribution of candidate resistance genes in the genome of *B*. *napus*

Homology and motif-based searches were used to discover candidate resistance genes that encode proteins with LRR domains in the genome of *B*. *napus*. The MEME Suite for motif discovery [25] was used to identify 261 eLRR genes. Whilst 174 of these have predicted transmembrane (TM) regions, 87 represent SPs. Using the *LepR3* sequence [13] for BLASTP added another 10 and 34 putative RLPs and SPs, respectively (S1 Table). NLRs had already been annotated and more recently analyzed in detail [5, 21].

**Fig 1:**
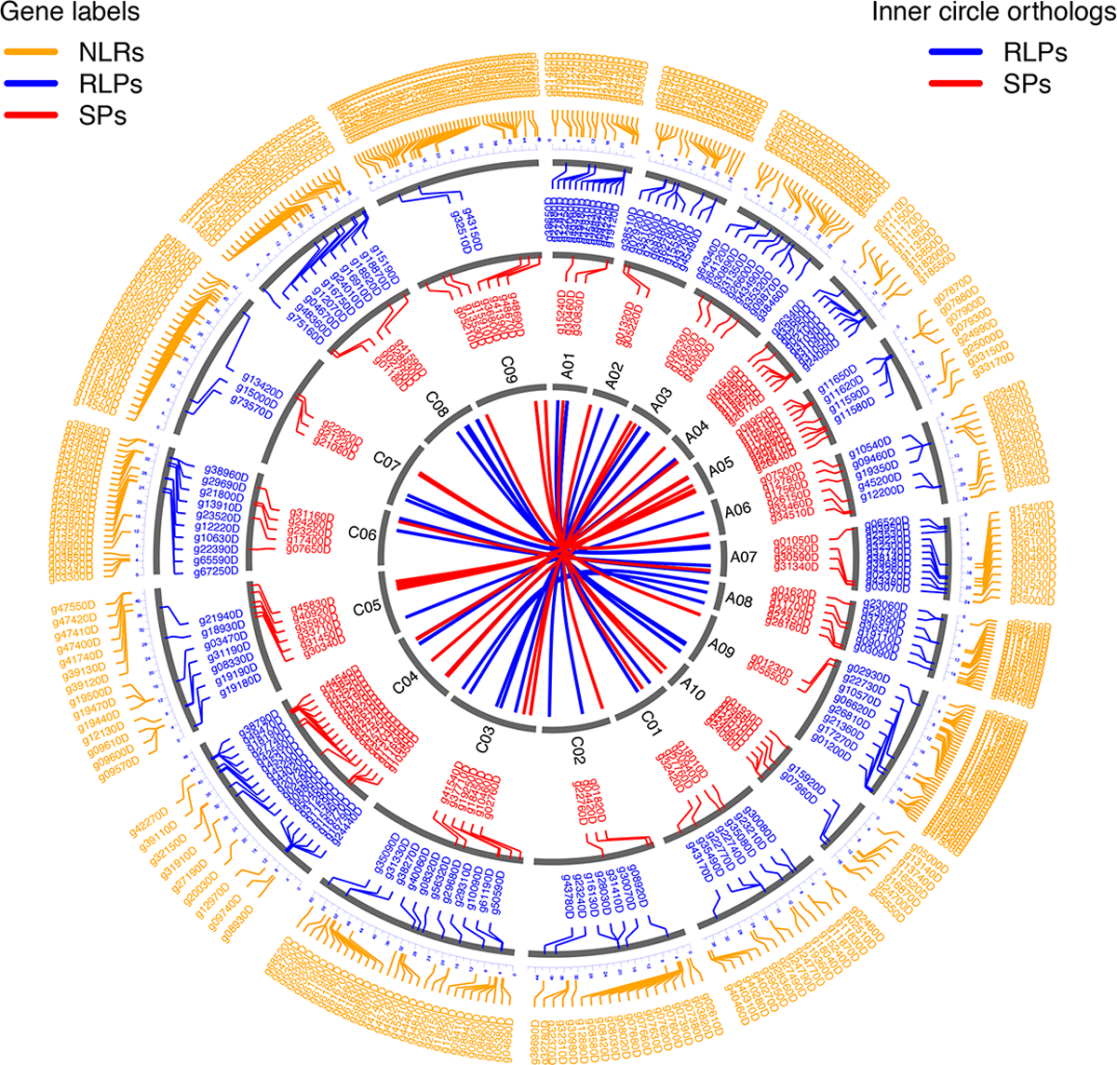
Distribution of candidate genes for resistance against pathogens in the genome of *Brassica napus*. All 19 (10 A and 9 C) chromosomes are displayed in grey as concentric circles according to the published *B*. *napus* genome sequence [21]. Genes encoding nucleotide-binding leucine-rich repeat receptors (NLRs), receptor-like proteins (RLPs) and secreted peptides (SPs) are color-coded in orange, blue and red, respectively. The center of this diagram shows homeologous relationships on the A and C sub-genomes between orthologous RLP and SP gene pairs in blue and red, respectively.

The genome-wide distribution of RLP, SP and NLR genes was visualized and determined as clustered (Fig 1). The distributions of homeologous pairs of RLP and SP genes were also tabulated and visualized (S2 Table and Fig 1). Pair-wise comparisons of the three gene families revealed that their relative distributions are not uniform (Table 1). Relative distances between these gene families were significantly positively correlated, indicating that they are close to one another on a genome scale (S3 Table). The spatial association between NLR and RLP genes was significant only when RLP query sequences were compared to NLR reference sequences, but not when NLR query and RLP reference sequences were compared. Similarly, evidence for close distances between eLRR and NLR genes was obtained only when they were used as query and reference sequences, respectively, but not the other way around. These findings show that spatial associations of eLRR or RLP genes are skewed towards NLR genes. In contrast, close association between SP genes and either RLP or NLR genes was significant in both directions. There is evidence for spatial overlap among these three gene families, although both Jaccard and projection tests were not significant at the genome scale. However, overlap was significant in pair-wise comparisons at the level of specific chromosomes (S3 Table). Although the Jaccard test provided no evidence for overlap between eLRR and NLR genes at the genome scale, the opposite was true at the chromosome scale with some of those overlaps being significant (S3 Table). Contrary results were obtained when all of these LRR genes were compared to ribosomal genes or to all predicted coding genes in the *B*. *napus* genome (Table 1). The positively correlated spatial distribution of these three gene families may reflect related gene function, regulation or evolution.

**Table 1:** Summary of statistical tests used to determine genome-wide spatial correlations between extracellular receptor-like proteins (RLPs) and secreted peptides (SPs) versus intracellular nucleotide-binding leucine-rich repeat receptors (NLRs).

	Query	Reference	Relative Kolmogorov-Smirnov *P*-value^a^	Relative ECDF area correlation^b^	Relative ECDF deviation area *P*-value			Jaccard measure *P*–value			Projection test *P*–value		
						Close^c^	Far		Overlap^d^	N		Overlap	N
eLRR vs. NLR genes^e^	256	366	3.2E-06	0.152	<0.01	✓	✓	0.43		✓	0.44	✓	
NLR vs. eLRR genes^e^	366	256	0.862	-2.2E-04	0.72		✓	0.40		✓	0.34	✓	
RLP vs. NLR genes	152	366	1.2E-04	0.141	0.01	✓		0.44	✓		0.29	✓	
NLR vs. RLP genes	366	152	0.007	0.046	0.07	✓		0.45	✓		0.24	✓	
SP vs. NLR genes	104	366	0.009	0.167	<0.01	✓		0.25	✓		0.21	✓	
NLR vs. SP genes	366	104	3.8E-04	0.064	0.02	✓		0.38	✓		0.14	✓	
SP vs. RLP genes	104	152	0.010	0.129	0.03	✓		0.12	✓		0.07	✓	
RLP vs. SP genes	152	104	8.2E-05	0.241	<0.01	✓		0.05	✓		0.06	✓	
LRR vs. ribosomal genes^e^	622	1134	0.002	-0.054	<0.01		✓	<0.01		✓	0.21		✓
Ribosomal vs. LRR genes^e^	1134	622	0.289	0.021	0.21	✓		<0.01		✓	0.02		✓
LRR vs. all other genes^e^	622	80305	0.000	-0.261	<0.01		✓	<0.01		✓	8.9E-78		✓
All other genes vs. LRR^e^	80305	622	2.4E-04	0.009	<0.01	✓		<0.01		✓	#######		✓

^a^ *P* values are shown for all tests in both directions, using one dataset as a query and the other one as a reference.

^b^ ECDF: Empirical Distribution Cumulative Function

^c^ For the relative distance test, positive and negative relative ECDF area correlation values are labeled as close and far, respectively.

^d^ The outcome of Jaccard and projection tests is defined as overlapping or non-overlapping (N).

^e^ The combination of RLP and SP genes are referred to as eLRR genes. Control comparisons include those of all predicted leucine-rich repeat (LRR) genes, consisting of RLP, NLR and SP genes, to ribosomal genes or those to all other coding genes of the *Brassica napus* genome.

### Pathogen-regulated expression of candidate resistance genes

**Fig 2:**
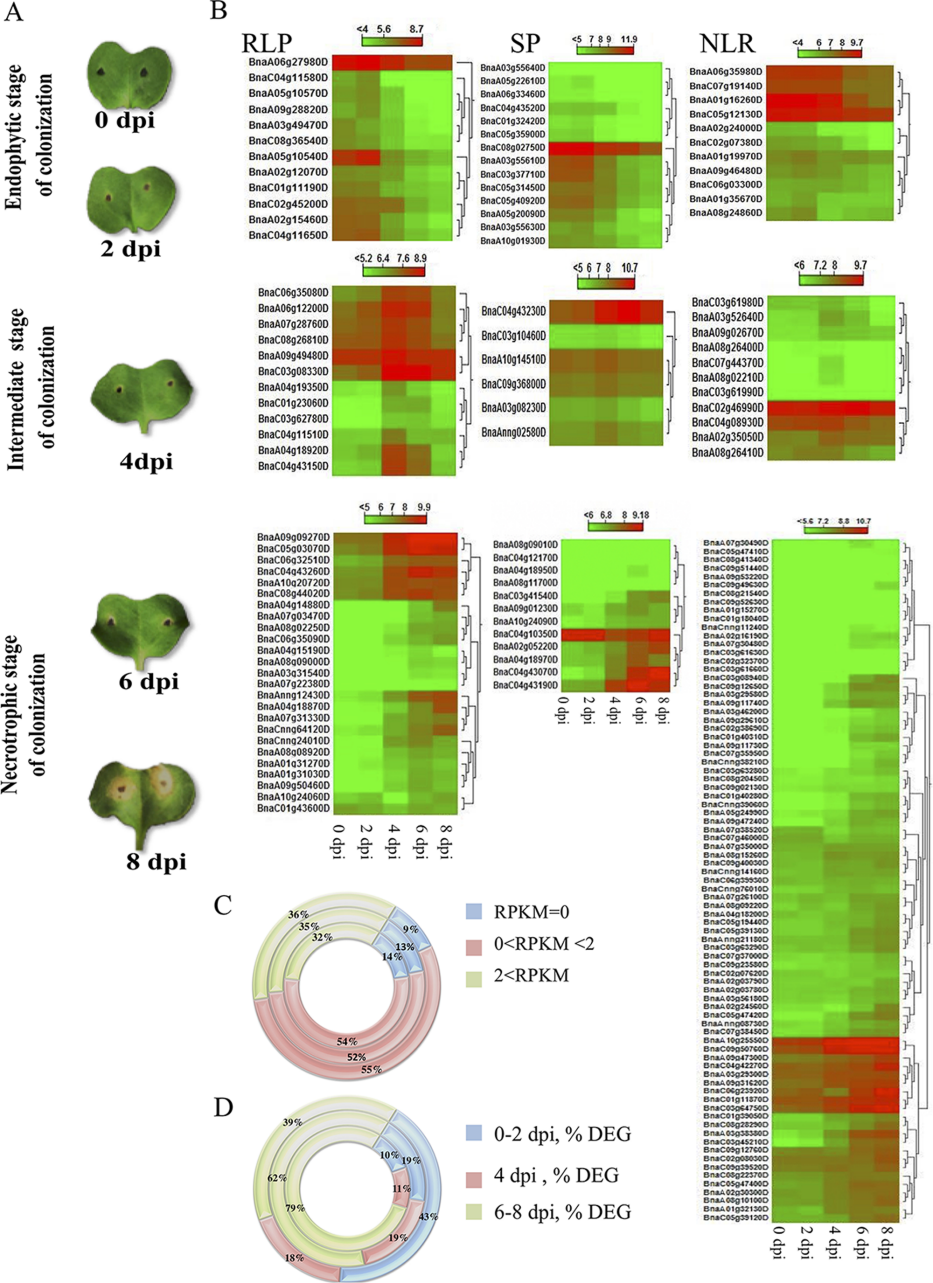
Symptoms and expression of candidate *R* genes after inoculation of susceptible *Brassica napus* cultivar Topas DH16516 with *Leptosphaeria maculans* isolate 00–100. (A) Appearance of cotyledons 0, 2, 4, 6 and 8 days post-inoculation (dpi) with *L*. *maculans*. (B) Heat maps of differentially expressed genes (DEG) encoding receptor-like proteins (RLPs), secreted peptides (SPs) and nucleotide-binding leucine-rich repeat receptors (NLRs). The expression of each gene is based on regularized logarithmic transformation (rld) of the average of the best three biological replicates. Expression patterns are grouped into initial endophytic (0–2 dpi), intermediate (4 dpi) and late necrotrophic (6–8 dpi) stages of colonization [22]. (C, D) Classification of different gene families into different expression categories. Circles (from inside to outside) represent NLR, RLP and SP genes. (C) General expression patterns, reads per kilobase million (RPKM), did not differ between RLP, SP and NLR genes (S4 Table, *χ*^*2*^ = 2.51, *P* = 0.64). (D) Percentages of differentially expressed genes (% DEG) at the three stages of colonization are shown. Induced expression patterns differed between RLP, SP and NLR genes (S5 Table, *χ*^*2*^ = 22.13, *P* = 0.0002).

The regulated mRNA expression of all genes belonging to RLP, SP or NLR gene families was analyzed after inoculation of the susceptible *B*. *napus* cultivar Topas with *L*. *maculans* [22]. Pathogen-induced mRNA expression patterns were grouped into three stages based on timing of their induction (Fig 2A, 2B and 2D). General uninduced mRNA expression was also grouped into three categories based on levels of expression (Fig 2C). The three gene families did not differ significantly in their uninduced mRNA expression levels (S4 Table). In contrast, the pattern of pathogen-induced mRNA expression differed significantly (S5 Table). Whereas the majority of SP genes were induced during the early endophytic phase, 0–2 days post-inoculation (dpi), induction of NLR gene expression occurred predominantly during the late necrotrophic phase of colonization (6–8 dpi). Induction of RLP gene expression was intermediate between these extremes (Fig 2). These differences in pathogen-induced mRNA expression may indicate that SP and NLR genes are important during early endophytic and late necrotrophic stages of colonization, respectively.

### LRR-containing candidate genes at two loci for resistance against *L*. *maculans*

Two loci for resistance against *L*. *maculans* that were considered are located on chromosome A07 of *B*. *napus*; a resistance cluster that includes *Rlm3*, *Rlm4*, *Rlm7* and *Rlm9* and the *Rlm1* locus at the bottom of chromosome A07. Each of these loci was examined for the presence of LRR genes. A single RLP gene, a homolog of the *A*. *thaliana* gene *TOO MANY MOUTHS* (*AtTMM*), was found in the resistance cluster that includes *Rlm7* and more narrowly *Rlm3* and *Rlm4* [26]. Two additional tandemly duplicated RLP genes and two NLR genes (S6 Table) were identified within the *Rlm9* locus that extends further towards the bottom of chromosome A07 [26]. Two RLP genes and one SP gene were discovered within the locus of *Rlm1* (Table 2).

**Table 2:** Test for evidence of positive selection in coding sequences of *Brassica napus R* genes and candidate genes for resistance against *Leptosphaeria maculans*.

Gene name^a^	Gene ID	Locus	Sequences^b^	Model^c^	d_N_/d_S_^d^	lnL^e^	χ^2^^f^	*P* value
*LepR3*/*Rlm2*	BnaA10g20720D	*LepR3*/*Rlm2*	4	Nearly neutral	0.658	-5028.4	18.49	0.00010
				Positive selection		-5019.1		
*LepR3*/*Rlm2*	BnaA10g20720D	*LepR3*/*Rlm2*	11	Nearly neutral	0.583	-3040.4	47.43	<0.00001
				Positive selection		-3016.7		
"Homeolog"	BnaN19g55980D	N/A^g^	3	Nearly neutral	0.598	-2195.1	1.18	0.554
				Positive selection		-2194.5		
*AtTMM*^h^	BnaA07g38270D	*Rlm4*, *Rlm9*	7	Nearly neutral	0.269	-3255.3	0.37	0.831
				Positive selection		-3255.1		
"TIR-NLR1"	BnaA07g22940D	*Rlm9*	7	Nearly neutral	0.743	-8074.3	24.70	<0.00001
				Positive selection		-8062.0		
"TIR-NLR2"	BnaA07g24260D	*Rlm9*	8	Nearly neutral	0.462	-3602.0	0.00	1.000
				Positive selection		-3602.0		
*AtRLP15*	BnaA07g22390D	*Rlm9*	12	Nearly neutral	0.482	-1056.9	0.56	0.756
				Positive selection		-1056.6		
*AtRLP12*	BnaA07g23530D	*Rlm9*	13	Nearly neutral	0.289	-257.3	0.18	0.914
				Positive selection		-257.3		
*AtCLV2*^h^	BnaA07g29310D	*Rlm1*	11	Nearly neutral	0.587	-3352.6	2.27	0.321
				Positive selection		-3350.4		
"BnRLP"	BnaA07g28760D	*Rlm1*	9	Nearly neutral	0.292	-4368.4	0.00	1.000
				Positive selection		-4368.4		
"BnSP"	BnaA07g28550D	*Rlm1*	6	Nearly neutral	0.384	-692.2	0.00	0.999
				Positive selection		-692.2		

^a^ Gene names refer to published *B*. *napus* names or *Arabidopsis thaliana* gene names; names in quotation marks merely refer to categories of genes.

^b^ Number of sequences analysed

^c^ Nearly neutral model parameters: ω_0_<1, ω_1_ = 1; positive selection model parameters: ω_0_<1, ω_1_ = 1, w_2_>1; ω is the ratio of non-synonymous to synonymous substitution rates (18).

^d^ Average non-synonymous to synonymous substitution rates.

^e^ lnL: log-likelihood

^f^ Likelihood ratio test: χ^2^ = 2 x (lnL_selection_—lnL_neutral_), df = 2.

^g^ Not applicable

^h^ *AtTMM* and *AtCLV2* are also annotated as *AtRLP17* and *AtRLP12*, respectively.

**Fig 3:**
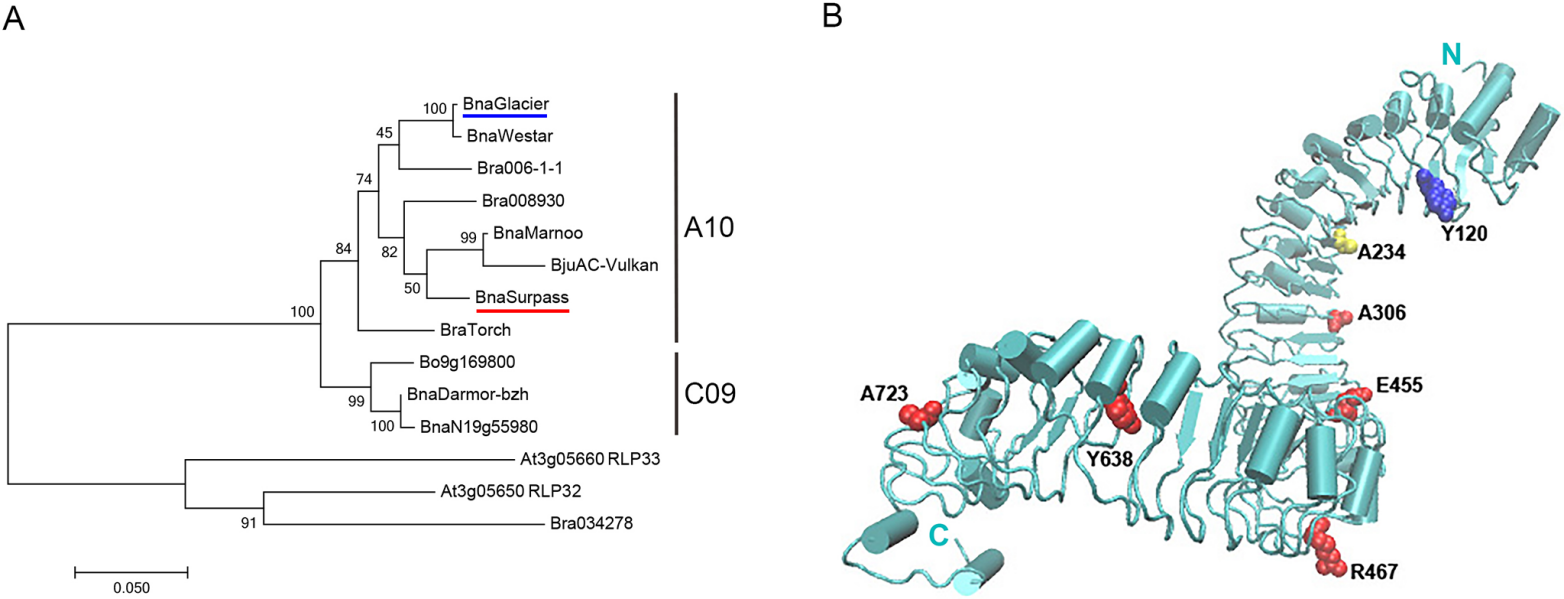
Phylogenetic tree and structural model of the leucine-rich repeat (LRR) domain of *Brassica napus* gene *LepR3* for resistance against *Leptosphaeria maculans*. (A) Maximum likelihood phylogenetic tree of genes belonging to the *LepR3*/*Rlm2* locus. Coding sequences were used to generate the tree. The Jukes-Cantor model [28] was used. The tree with the greatest log-likelihood is shown. Numbers indicate bootstrap values of 1,000 replicates. A discrete Gamma distribution was used to model evolutionary rate differences between sites. The rate variation model allowed for some sites to be evolutionarily invariable. Branch lengths are measured as the number of substitutions per site (scale bar). Branches designate species abbreviations: Bna = *Brassica napus*, Bra = *B*. *rapa* and Bju = *B*. *juncea*, followed by cultivar names or IDs, or accession numbers; BnaGlacier: AJG42078, BnaWestar: AJG42083, Bra 006-1-1: AJG42088, Bna-Marnoo: AJG42089, BjuAC-Vulkan: AJG42090, BnaSurpass: AGC13588, BraTorch: AJG42087. Gene IDs for genes that encode a receptor-like protein (RLP) are given after *Arabidopsis thaliana* accession numbers. Sequences from *B*. *napus* cultivars Glacier and Surpass, underlined in blue and red, respectively, have *Rlm2* and *LepR3* alleles, respectively, of the resistance gene on chromosome A10. Alleles identical to those from Glacier and Surpass were sequenced from three and two different cultivars, respectively. The sequence of cultivar Westar (no known *R* gene against *L*. *maculans*) was identical to sequences from eight other cultivars. (B) The modeled LRR domain starts at amino acid 27 and ends at amino acid 804. Seven amino acids predicted to be under positive selection are shown as vdW spheres. Residues in yellow (A234) and red (A306, E455, R467, Y638, A723) are supported at the 95% and 99% confidence level, respectively, using a comparison of 11 coding sequences. The residue in blue (Y120) is supported at the 95% level using four coding sequences.

To predict a possible involvement of these candidate eLRR genes in resistance against *L*. *maculans*, phylogenetic analysis by maximum likelihood (PAML) was done. *LepR3* and *Rlm2* were used as references; both are alleles of the same *R* gene that are effective against different races of *L*. *maculans*. A phylogenetic comparison is shown between *LepR3* and *Rlm2* resistance alleles, orthologous genes from *B*. *napus*, *B*. *rapa* and *Brassica juncea* (chromosome A10 group), homeologous genes from *B*. *napus* and *Brassica oleracea* (chromosome C09 group) and paralogous genes from *A*. *thaliana* and *B*. *napus* (Fig 3A). A model that included positive selection fitted the data significantly better than a nearly neutral model (Table 2). This was the case even when *LepR3* and *Rlm2* alleles were excluded from a dataset of three orthologous sequences. By contrast, there was no evidence for positive selection of a homeologous gene on chromosome C09 (BnaN19g55980D), which has not been suggested to be involved in resistance against pathogens. The function of BnaN19g55980D is therefore probably different from that of BnaA10g20720D, *LepR3* and *Rlm2*.

Similar phylogenetic comparisons and corresponding sequence alignments were used to determine selection of candidate *R* genes at the two resistance loci on chromosome A07. There was no evidence for positive selection of BnaA07g38270D, the *B*. *napus* homolog of *AtTMM*, which was the only LRR gene identified at the *Rlm3*-*Rlm4*-*Rlm7* resistance cluster (Table 2). As the cv. DH12075 genome sequence used contains *Rlm4*, LRR genes are probably not responsible for resistance against corresponding avirulent *L*. *maculans* races with *AvrLm4*. Among the recognized LRR genes at the *Rlm9* locus, only BnaA07g22940D, a putative TIR-NLR, was positively selected. As the cv. Darmor *bzh* genome sequence used contains *Rlm9*, it is possible that BnaA07g22940D confers resistance against corresponding *L*. *maculans* races with *AvrLm5-9* [27]. Whereas chromosome C06 homeologs from three *B*. *napus* cultivars belonged to the same clade, the putative cv. ZS11 (XM_013844543) ortholog on chromosome A07 did not group with BnaA07g22940D and the corresponding cv. DH12075 gene (BnaN07g24870), demonstrating that evolution at these two loci is different (S1 Fig). Three eLRR genes at the *Rlm1* locus were not positively selected. A genome sequence that contains *Rlm1* was not used in this study and it is therefore possible that another LRR gene not present in cv. Darmor *bzh* and cv. DH12075 genomes is responsible for resistance against *L*. *maculans* races with *AvrLm1*.

Posterior probabilities of the PAML analysis were used to identify positively selected amino acids for *LepR3* or *Rlm2*. A predicted protein structure was used to visualize these positively selected amino acids and six out of seven were surface exposed (Fig 3B). Moreover, the four N-terminal amino acids lined a concave surface of the predicted molecule and together may be involved in interacting with a target protein.

### Linkage of candidate genes to resistance against intracellular obligate biotrophic, extracellular hemi-biotrophic and necrotrophic pathogens

**Fig 4:**
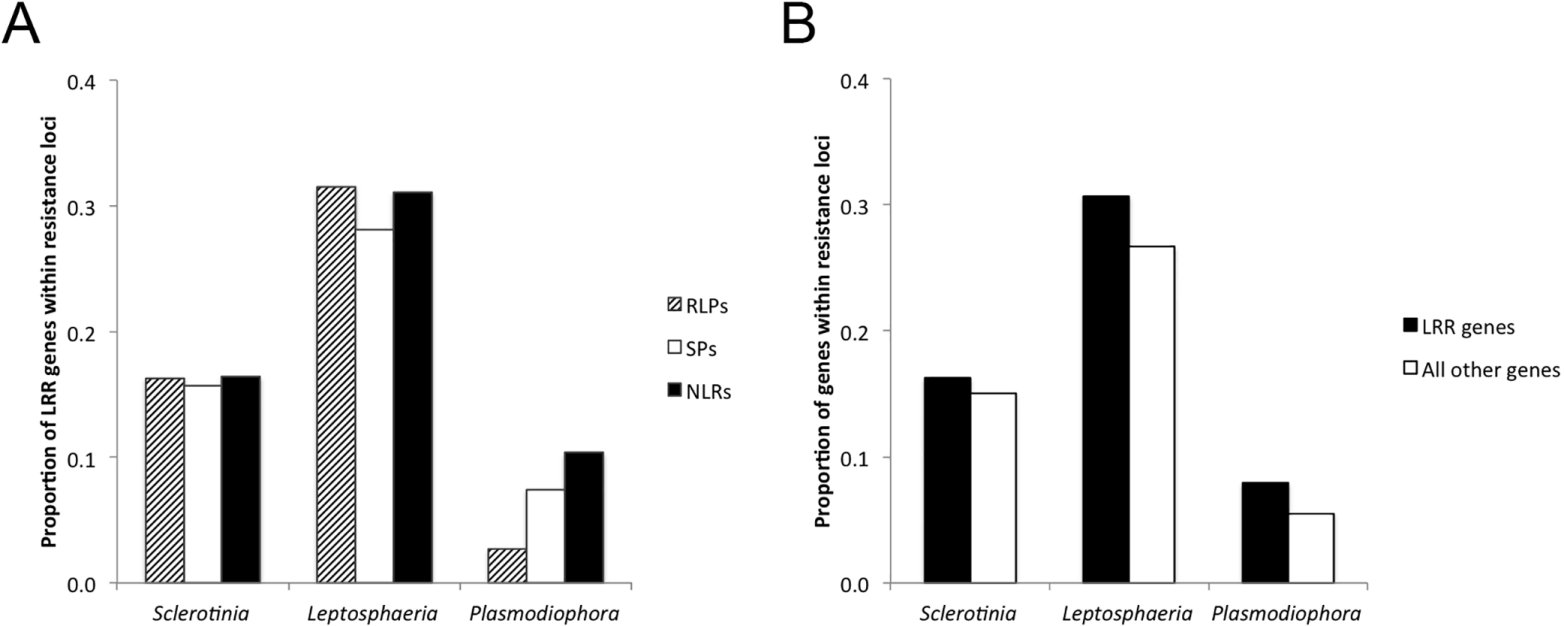
Proportions of *Brassica napus* genes encoding receptor-like proteins (RLPs), secreted peptides (SPs) or nucleotide-binding leucine-rich repeat receptors (NLRs) within chromosomal intervals (loci) for resistance against *Sclerotinia sclero*tiorum (necrotroph), *Leptosphaeria maculans* (extracellular) or *Plasmodiophora brassicae* (intracellular). (A) Proportions represent the numbers of RLP, SP or NLR genes within the mapped regions for resistance against *S*. *sclero*tiorum, *L*. *maculans* or *P*. *brassicae* divided by the total number of genes for each of the three families. Contingency tests show that proportions of RLP, SP and NLR genes are not equal for resistance against *P*. *brassicae* (*χ*^*2*^ = 9.84, *P* = 0.007). (B) Proportions represent the total number of LRR genes (RLP, SP and NLR) within mapped regions for resistance divided by the total number of LRR genes (black bars). These proportions were compared to those for all of the genes within mapped regions for resistance divided by the total number of genes within the genome (white bars). Differences between these two proportions were not significant (contingency table, *χ*^*2*^ = 1.90, *P* = 0.39).

Published loci for resistance against three pathogens with different life styles, *S*. *sclerotiorum* (necrotrophic), *L*. *maculans* (extracellular, hemibiotrophic) and *P*. *brassicae* (intracellular, obligate biotrophic), were considered to determine any association with RLP, SP or NLR gene families. A list of all RLP, SP and NLR genes found within specific resistance loci was generated (S6 Table). The sums of all unique genes belonging to each gene family and found to be positioned at these loci for resistance against a particular pathogen were generated and subsequently analyzed. A significant over-representation of NLR genes was detected for resistance against the obligate intracellular biotrophic protist *P*. *brassicae* (Fig 4A). In contrast, the proportional representation of RLP, SP and NLR genes was similar when resistance against the necrotrophic pathogen *S*. *sclerotiorum* or the extracellular hemibiotrophic pathogen *L*. *maculans* was considered. Of note, loci for resistance against *L*. *maculans* included both *R* genes and quantitative trait loci (QTL). LRR genes were not significantly over-represented in loci for resistance against these three pathogens (Fig 4B).

## Discussion

Protein motif and homology searches were used to determine and analyze the distribution of LRR genes encoding NLRs, RLPs and SPs in the genome of *B*. *napus*. Domains/motifs were used to generate the RGAugury pipeline for identification of four resistance gene analog (RGA) families encoding NLRs, RLPs, RLKs and proteins with transmembrane and coiled-coil domains [5]. RGAugury data on the *B*. *rapa* genome, i.e. 244 NLR, 118 RLP and 747 RLK genes [5], compares favorably with 415 NLR, 184 RLP and 938 RLK genes reported here, taking into account the larger genome of *B*. *napus*, containing A and C sub-genomes corresponding to the genomes of *B*. *rapa* and *B*. *oleracea*, respectively.

Emphasis was on three globally important pathogens that differ in their infection and colonization strategies. Evidence was obtained that NLR genes control intracellular plant pathogens. Even though 29 quantitative and qualitative loci for resistance against the obligate biotrophic protist *P*. *brassicae* were considered, a significant enrichment of NLR genes was obtained (Fig 4A and S6 Table). This finding confirms, at the genome scale, the importance of NLR genes in resistance against *P*. *brassicae* previously indicated through cloning of the *Crr1a* gene [15] and fine-mapping and the *Rcr1* locus [17]. Although two map locations were used for each of the *CRa* and *Crr1* loci (S6 Table), this did not create a bias because only unique LRR genes were analyzed (Fig 4A). It is therefore possible that most genes for resistance against *P*. *brassicae* will encode NLRs [29, 30].

Evidence for an involvement of eLRR genes in resistance against the extracellular pathogen *L*. *maculans* was less obvious. A rational assumption may be that an *R* gene that encodes an LRR protein is under positive selection [23, 24]. This assumption was supported by the fact that the RLP gene that confers *LepR3* and *Rlm2* resistance specificities is under positive selection (Table 2). Only one of eight tested candidate *R* genes within the two resistance loci on chromosome A07, the TIR-NLR gene BnaA07g22940, was positively selected, suggesting that this TIR-NLR may represent *Rlm9*. It seems unlikely that the *BnTMM* gene (BnaA07g38270D) is a candidate for *Rlm4*, although this gene is found at different locations of the cv. Darmor-*bzh* and DH12075 genomes. Other TM-receptors not containing eLRR domains may be suitable candidate resistance genes [31]; this may not be unexpected because the recently described *Rlm12* gene could encode a cysteine-rich kinase [32, 33].

Resistance against *L*. *maculans* was also analyzed at the genome scale considering a total of 99 qualitative and quantitative trait loci (S6 Table). None of the three LRR genes families was enriched in loci for resistance against *L*. *maculans* (Fig 4A), suggesting that different types of genes may contribute to resistance against this pathogen. This notion is supported by the finding that a positively selected TIR-NLR gene is present at the *Rlm9* locus. As with *P*. *brassicae*, both *R* gene and quantitative resistance loci were included in this analysis of *L*. *maculans*.

Gene expression was analyzed in cv. Topas with sequenced reads mapped back to the reference genome of cv. Darmor *bzh* [21, 22]. Pathogen-induced expression differed significantly between RLP, SP and NLR gene families. SP and NLR genes were expressed early and late during colonization of *B*. *napus* by *L*. *maculans*, respectively (Fig 2). The temporally less defined pathogen-induced expression of RLP genes may be due to the fact that some members are involved in developmental processes while others relate to resistance against pathogens. The relatively late expression of most NLR genes (6–8 dpi) makes it less likely that this gene family is intimately involved in regulation of the early endophytic phase of fungal colonization. Of note, the positively selected TIR-NLR gene, BnaA07g22940D located within the *Rlm9* locus, was expressed at low levels and not regulated by *L*. *maculans*. In contrast, the *B*. *napus* ortholog of *AtRLP30* (BnaA06g12200D) was expressed during the intermediate phase (4 dpi) of pathogen colonization (Fig 2A). This gene was also recently shown to be expressed earlier (3 dpi) during an incompatible interaction between *B*. *napus* and *L*. *maculans* [10]. The ortholog of *LepR3*/*Rlm2*, BnaA10g20720D (Table 2), was induced late during the compatible interaction of *L*. *maculans* with cv. Topas (Fig 2).

With respect to quantitative resistance against the necrotrophic pathogen *S*. *sclerotiorum*, none of the three LRR gene families was enriched (Fig 4). Whilst these data do not imply that LRR genes are unimportant, it may not be expected that LRR genes feature strongly in resistance against *S*. *sclerotiorum* because there is currently no evidence that *R* genes control this pathogen [34]. Moreover, expression of the SP gene *BnPGIP2* did not confer long-term resistance against *S*. *sclerotiorum* in transformed *B*. *napus* plants [35].

Clustering of candidate resistance genes in the genome of *B*. *napus* (Fig 1) confirms previous findings about NLR genes [21]. Clusters of *R* genes encoding RLPs have also been reported [36]. Less is known about SPs, although clustering of SPs with eLRR domains does occur in animals [37]. The correlated distribution of these three gene families in the *B*. *napus* genome (Table 1) is intriguing and may have been selected through evolution. Close association of LRR genes may have consequences for coordinated regulation of gene expression or sequence exchanges through unequal cross-overs or gene conversion, not only within a gene family but also between gene families. General gene expression did not differ among the three gene families (Fig 2), suggesting that this may be a reflection of their correlated genome distribution (Table 1). Sequence exchanges between eLRR genes are considered more likely because adjacent pairs of genes were observed only between those coding for RLPs or SPs (S1 Table). All eLRR genes may therefore comprise a single superfamily of genes [37]. Additional analysis of 58 homeologous pairs of RLP and SP genes recognized 14 out of 21 homeologous regions identified previously, based on the relationship of A and C sub-genomes in the cv. Darmor *bzh* reference genome sequence [21].

The findings reported here will help the *Brassica* research and breeding communities to identify genes controlling agronomic traits, such as defense against pathogens and plant development. Research and breeding efforts should not solely be focused on LRR genes, especially in the case of resistance against *L*. *maculans* and *S*. *sclerotiorum* (Fig 4). Unbiased genome-wide association studies of quantitative resistance traits against several pathogens using a diverse panel of *B*. *napus* accessions [6] will identify novel resistance genes. Although Arabidopsis studies show that quantitative resistance involves a complex defense network consisting of over a thousand genes [38], the LRR genes reported here will be invaluable for generating markers to map and clone genes involved in resistance against pathogens, following proven approaches to exploit highly polymorphic LRR domains for isolation of resistance genes [38, 39].

## Materials and methods

### Plant growth and pathogen inoculation

The susceptible *B*. *napus* doubled-haploid (DH) line Topas DH16516 and a single-spore culture of the *L*. *maculans* isolate 00–100 with well-characterised avirulence interactions (*AvrLm2*, *AvrLm3*, *AvrLm6*, *AvrLm9*, *AvrLmS*, *AvrLep1*, *AvrLep2* and *AvrLep4*) were used [22]. Plants were grown in a growth chamber at 20°C, 16 h day length, with a light intensity of c. 450 μmol m^−2^ s^−1^ and at 18°C for 8 h darkness. For conidial inoculation, a small wound was made in the center of each cotyledon of 7-day-old seedlings and 10μL of 2×10^7^ spores/mL suspension was applied to each wound. After inoculation, the seedlings were kept under the same growth conditions and symptom development was monitored.

### Analysis of Illumina^®^ sequence reads

Illumina^**®**^-based sequence reads were mapped back to the sequenced cv. Darmor *bzh* genome [21], as previously described [22]. Despite the use of this most comprehensive and available genome [21], it cannot be excluded that few genes may be missed in the absence of a sequenced and annotated cv. Topas genome.

### Motif and homology searches for transmembrane and extracellular LRR receptors

The *de novo* motif discovery tool of the MEME algorithm [25] recognized 20 motifs with a width of ≤100 amino acids from 57 known *A*. *thaliana* RLP sequences [9] (S7 Table). All 20 motifs were used to search the *B*. *napus* genome for RLPs using MAST [25]. A total of 1249 sequences were identified (*E*-value <0.01, position *P*-value <0.0001). Domains of candidate sequences were predicted using Pfam and sequences with kinase domains were eliminated, resulting in 311 RLP candidates. All of these sequences were checked using the prediction servers TMHMM, applying a hidden Markov model, [40] and Phobius [41] for presence of signal peptides (SignalP) and/or transmembrane (TM) regions; Pfam [42] and SMART [43] prediction servers were used to identify LRR domains. LRR proteins that were predicted to contain neither TM regions nor SignalP were eliminated. Of the final 261 sequences, 174 were predicted to contain TM regions.

The predicted eLRR domain of *LepR3* [13] was used for BLASTP against the genome of *B*. *rapa*, resulting in a total of 444 putative RLP sequences. Pfam was used to reduce this list to 239 sequences by eliminating genes encoding kinase domains. BioMart of Ensemble plants was used to eliminate genes lacking LRR domains, SignalP and/or TM regions, resulting in a final list of 167 genes. *B*. *napus* orthologs were identified using a published synteny map [21]. Pfam reduced the list of *B*. *napus* sequences containing LRR domains to 76. Prediction of transmembrane domains with TMHMM and Phobius revealed a final 10 putative RLP sequences and 34 secreted LRR proteins (S1 Table).

### Genome-wide distribution and statistical analysis of genes with LRR domains

The distribution of candidate resistance genes was visualized using the R package OmicCircos [44]. The R package GenometriCorr was used to determine whether the different NLR, RLP and SP genes were spatially correlated [45].

### Gene expression analysis

An RNAseq dataset [22] was used to evaluate the expression of candidate resistance genes. Uninduced and pathogen-induced expression data were compared among RLP, SP and NLR genes. Contingency tables were generated and *Χ*^*2*^-tests were done in R to determine significant differences in gene regulation among these three gene families.

### Physical locations for loci for resistance against *L*. *maculans*, *P*. *brassicae* or *S*. *sclerotiorum*

Sequences of markers from previously published resistance loci were used to define physical intervals within the *B*. *napus* cv. Darmor-*bzh* reference genome via BLAT using the Genoscope web portal [32]. Where full intervals could not be defined, a region spanning 1 Mb either side of single linked markers was used. Where genomic intervals were already defined for the *B*. *rapa* or *B*. *oleracea* genomes, the nearest genes to each end of the intervals were used for alignment to the *B*. *napus* genome.

### Phylogenetic analysis of candidate *R* genes

Homologs of RLP and SP genes were identified in sequenced genomes of *B*. *napus*, *B*. *rapa*, *Brassica oleracea*, *Brassica juncea*, *A*. *thaliana* and *Capsella rubella*. In addition to released genome sequences, the genome sequence of *B*. *napus* cv. DH12075 (courtesy of Dr. Isobel Parkin, AAFC Saskatoon) was used; this is available on request [4]. Its sequence information can be accessed; BioProject ID: PRJNA218846 [3]. MEGA7 [46] was used to generate maximum likelihood (ML) phylogenetic trees (S1 Appendix). Specifically, coding sequences were translated and aligned before bootstrap analysis. Tree and alignment files were generated and used in Phylogenetic Analysis by ML program package [47]. CODEML was used to compare different models, including a nearly neutral model with a ratio of nonsynonymous/synonymous substitution rates (*ω* = *d*_*N*_/*d*_*S*_) of *ω*_0_ < 1 and *ω*_1_ = 1 and a positive selection model with an additional condition of *ω*_2_ > 1. Likelihood ratio tests and posterior probabilities were computed. Bayes empirical Bayes (BEB) estimates were used to identify positively selected amino acid residues.

### Structural modeling of the *LepR3* LRR domain

The LRR domain of *LepR3* was used for BLASTP against each of the plant genomes within the Ensemble database. Sequences with an *E*-value < 10^−10^ were selected. CD-HIT was used to reduce redundancy with a sequence identity cut-off of 90% [48]. A total of 7663 sequences, 105–1195 amino acids in length, were aligned by running three iterations of Clustal Omega on a Linux server. EVfold [49] used a final alignment of 4740 sequences to compile a list of amino acid contacts. A total of 200 amino acid pairs with coupling scores of 0.075 to 0.296 and the primary amino acid sequence were entered in I-TASSER [50] for protein structure modeling. The pdb file of the best model was used for visualization in VMD [51].

### Detection of candidate gene hits within resistance loci

The R package GenomicRanges [52] was used to identify RLP, SP and NLR genes within specified chromosomal intervals of mapped resistance genes. A code was developed in R (S2 Appendix) that allowed automatic entry of genes within these intervals into S6 Table. Contingency tables were generated and *χ*^*2*^ analysis was done in R.

## Supporting information

S1 Table List of predicted leucine-rich repeat (LRR) receptors lacking kinase domains in the genome of *Brassica napus*. (ZIP)

S2 Table Homeologous pairs of genes on A and C chromosomes. (ZIP)

S3 Table Raw data output for pair-wise comparisons of candidate resistance genes using the CentometriCorr package in R [Favorov et al. (2012) PLOS Comp. Biol. 8, e1002529]. If overlaps between query and reference features occur more often than expected, the projection.test.lower.tail and jaccard.measure.lower.tail are FALSE; if they are less common than expected, they are TRUE. The projection.test.p.value is based on a two-sided binomial test. The jaccard.measure.p.value is based on permutation tests.
(XLSX)

S4 Table Contingency table of uninduced gene expression of nucleotide-binding leucine-rich repeat receptor (NLR), receptor-like protein (RLP) and secreted protein (SP) genes. (ZIP)

S5 Table Contingency table of pathogen-induced gene expression of nucleotide-binding leucine-rich repeat receptor (NLR), receptor-like protein (RLP) and secreted protein (SP) genes. (ZIP)

S6 Table Genes encoding nucleotide-binding leucine-rich repeat receptors (NLR), receptor like proteins (RLP) or secreted peptides (SP) at map locations of loci for resistance of *Brassica* spp. against *Sclerotinia sclerotiorum*, *Leptosphaeria maculans* or *Plasmodiophora brassicae*. (ZIP)

S7 Table Identification of amino acid sequence motifs among 57 amino acid sequences for receptor-like proteins from *Arabidopsis thaliana* (Wang et al., 2008). Sequences were discovered using the motif discovery tool MEME (Bailey et al., 2009).
(ZIP)

S1 Fig Maximum likelihood phylogenetic tree of *Brassica napus* sequences related to BnaA07g22940D. Coding sequences were used to generate the tree. The Jukes-Cantor model [26] was used. The tree with the greatest log-likelihood is shown. Numbers indicate bootstrap values of 1,000 replicates. A discrete Gamma distribution was used to model evolutionary rate differences between sites. The rate variation model allowed for some sites to be evolutionarily invariable. Branch lengths are measured in as the number of substitutions per site (scale bar). Branches designate species abbreviations: Bna = *Brassica napus*, Bra = *B*. *rapa* and Bo = *B*. *oleracea*, AT = *Arabidopsis thaliana* representing accession numbers except for accession numbers that start with XM from *B*. *napus* cv. ZS11; XM013843043.1 and XM013844543.2 are on chromosomes C06 (blue) and A07 (red), respectively. The line for chromosome A07 is broken because the *B*. *rapa* ortholog is included.
(ZIP)

S1 Appendix Tree files, specifying genes used for phylogenetic analysis by maximum likelihood. (ZIP)

S2 Appendix R code to generate hit tables based on published mapping information. The package GenomicRanges was used to compare genomic intervals of genes and resistance loci. Results were subsequently automatically inserted into pre-existing tables.
(ZIP)
