# Correction: Genomic evidence for genes encoding leucine-rich repeat receptors linked to resistance against the eukaryotic extra- and intracellular *Brassica napus* pathogens *Leptosphaeria maculans* and *Plasmodiophora brassicae*

**DOI:** 10.1371/journal.pone.0202143

**Published:** 2018-08-07

**Authors:** The PLOS ONE Journal Staff

In Table 1, the value in the row titled “All other genes vs. LRR^e^” and the column titled “Projection test *P*- value” should read as 1.9E-120. Please see the corrected Table 1 here. The publisher apologizes for this error.

**Table 1:** Summary of statistical tests used to determine genome-wide spatial correlations between extracellular receptor-like proteins (RLPs) and secreted peptides (SPs) versus intracellular nucleotide-binding leucine-rich repeat receptors (NLRs).

	Query	Reference	Relative Kolmogorov-Smirnov *P*-value^a^	Relative ECDF area correlation^b^	Relative ECDF deviation area *P*-value			Jaccard measure *P*–value			Projection test *P*–value		
						Close^c^	Far		Overlap^d^	N		Overlap	N
eLRR vs. NLR genes^e^	256	366	3.2E-06	0.152	<0.01	**✓**		0.43		**✓**	0.44	**✓**	
NLR vs. eLRR genes^e^	366	256	0.862	-2.2E-04	0.72		**✓**	0.40		**✓**	0.34	**✓**	
RLP vs. NLR genes	152	366	1.2E-04	0.141	0.01	**✓**		0.44	**✓**		0.29	**✓**	
NLR vs. RLP genes	366	152	0.007	0.046	0.07	**✓**		0.45	**✓**		0.24	**✓**	
SP vs. NLR genes	104	366	0.009	0.167	<0.01	**✓**		0.25	**✓**		0.21	**✓**	
NLR vs. SP genes	366	104	3.8E-04	0.064	0.02	**✓**		0.38	**✓**		0.14	**✓**	
SP vs. RLP genes	104	152	0.010	0.129	0.03	**✓**		0.12	**✓**		0.07	**✓**	
RLP vs. SP genes	152	104	8.2E-05	0.241	<0.01	**✓**		0.05	**✓**		0.06	**✓**	
LRR vs. ribosomal genes^e^	622	1134	0.002	-0.054	<0.01		**✓**	<0.01		**✓**	0.21		**✓**
Ribosomal vs. LRR genes^e^	1134	622	0.289	0.021	0.21	**✓**		<0.01		**✓**	0.02		**✓**
LRR vs. all other genes^e^	622	80305	0.000	-0.261	<0.01		**✓**	<0.01		**✓**	8.9E-78		**✓**
All other genes vs. LRR^e^	80305	622	2.4E-04	0.009	<0.01	**✓**		<0.01		**✓**	1.9E-120		**✓**

^a^ *P* values are shown for all tests in both directions, using one dataset as a query and the other one as a reference.

^b^ ECDF: Empirical Distribution Cumulative Function

^c^ For the relative distance test, positive and negative relative ECDF area correlation values are labeled as close and far, respectively.

^d^ The outcome of Jaccard and projection tests is defined as overlapping or non-overlapping (N).

^e^ The combination of RLP and SP genes are referred to as eLRR genes. Control comparisons include those of all predicted leucine-rich repeat (LRR) genes, consisting of RLP, NLR and SP genes, to ribosomal genes or those to all other coding genes of the *Brassica napus* genome.
