# Supplementary figures and images for: Genomic evidence for genes encoding leucine-rich repeat receptors linked to resistance against the eukaryotic extra- and intracellular Brassica napus pathogens Leptosphaeria maculans and Plasmodiophora brassicae
Source: PLoS One. 2018 Jun 1;13(6):e0198201. doi: 10.1371/journal.pone.0198201 (PMC5983482; doi:10.1371/journal.pone.0198201)

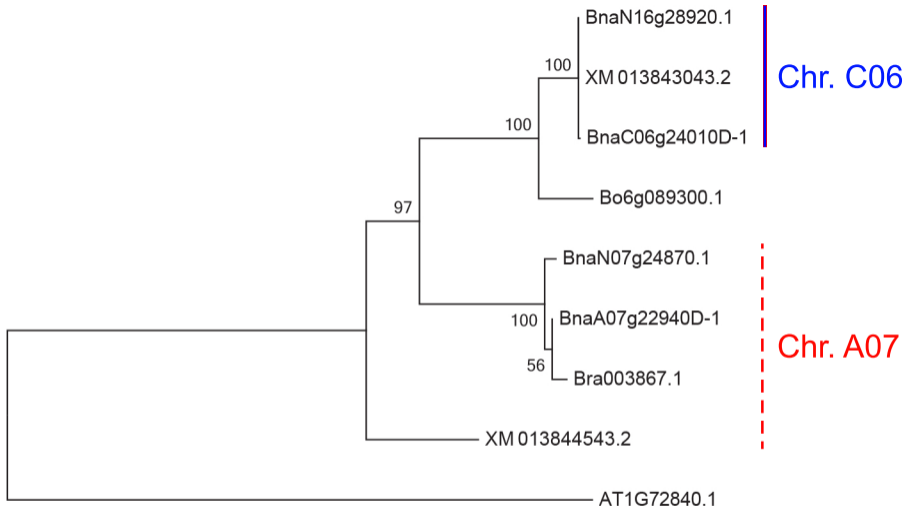

Supplement: S1 Fig — Coding sequences were used to generate the tree. The Jukes-Cantor model [26] was used. The tree with the greatest log-likelihood is shown. Numbers indicate bootstrap values of 1,000 replicates. A discrete Gamma distribution was used to model evolutionary rate differences between sites. The rate variation model allowed for some sites to be evolutionarily invariable. Branch lengths are measured in as the number of substitutions per site (scale bar). Branches designate species abbreviations: Bna = Brassica napus, Bra = B. rapa and Bo = B. oleracea, AT = Arabidopsis thaliana representing accession numbers except for accession numbers that start with XM from B. napus cv. ZS11; XM013843043.1 and XM013844543.2 are on chromosomes C06 (blue) and A07 (red), respectively. The line for chromosome A07 is broken because the B. rapa ortholog is included. (ZIP) [file pone.0198201.s008.zip › S1Figure.pdf]
