# Supplementary material for: Genomic evidence for genes encoding leucine-rich repeat receptors linked to resistance against the eukaryotic extra- and intracellular Brassica napus pathogens Leptosphaeria maculans and Plasmodiophora brassicae
Source: PLoS One. 2018 Jun 1;13(6):e0198201. doi: 10.1371/journal.pone.0198201 (PMC5983482; doi:10.1371/journal.pone.0198201)
Supplement: S1 Appendix — (ZIP) [file pone.0198201.s009.zip › S1Appendix-TreeFiles.docx]

**S1 Appendix. Tree files, specifying genes used for phylogenetic analysis by maximum likelihood.**

LepR3/Rlm2 tree:

(Bra008930,(BnaSurpass_JX880110,(BnaMarnoo_KM097079,BjuAC-Vulkan_KM097080)),((Bra006-1-1_KM097078,(BnaWestar_KM097073,BnaGlacier_KM097068)),(BraTorch_KM097077,(Bo9g169800,(BnaDarmor-bzh_ BnaCnng65590D,BnaN19g55980)))));

LepR3/Rlm2 small tree:

(BnaWestar_KM097073,BnaRlm2_Glacier_KM097068,(BnaSurpass_JX880110,BraTorch_KM097077));

LepR3 ortholog tree:

(DarmorBzh_BnaA10g20720D,BnaN10g22130,Bra008930);

LepR3 homeolog tree:

(BnaDarmor-bzh_ BnaCnng65590D,BnaN19g55980,Bo9g169800);

BnaA07g28760 tree

(((BnaA07g28760D,BnaN07g30740),Bra016226),(Bo6g110900,(BnaN16g37110,BnaC06g31350D)),(AT1G56130,(Carubv10008172m.g,AT1G56120)));

RLP12 tree

((((((BnaA07g29310D,BnaN07g31590),Bna_ZS11_RLP12),BjRLP12),(BnaC06g32510D,Bo6g112980)),Bra016141),AT1G71400_AtRLP12,(Bra016142,(BnaC06g43780D,Bo6g112940)));

SP tree

((BnaA07g28550D,BnaN07g30560),Bra016239,(Bo6g110730,(BnaC06g31160D,BnaN16g36950)));

TMMtree

(((BnaA07g38270D,BnaN07g21620),Bra003549),(Bo6g080150,(BnaC06g43050D,BnaN16g23990)),At1g80080);

TIR-NLR1 tree

(((BnaN07g24870.1,BnaA07g22940D),Bra003867),(Bo6g089300,(BnaN16g28920,BnaC06g24010D)),AT1G72840);

TIR-NLR2 tree

((((Bra003997,BnaN07g26180),BnaA07g24260D),BnaN16g31030),(BnaC06g25350D,Bo6g095450),(Alyrata_scaffold_0007_3919,AT1G69550));

AtRLP15 tree

(((((((Bo6g087200,BnaN16g28000),BnaC06g23240D),AT1G74190),BnaC06g23210D),(BnaA07g22370D,(Bra003815,BnaN07g24340))),(BnaA07g22390D,Bra003816)),AT1G74200,BnaA07g22380D);

AtRLP12 tree

((((((((Bo6g094240,Bra003925),BnaCnng07960D),BnaA07g23520D),BnaN16g29840),AT1G71400_RLP12),(BnaA07g23530D,BnaN07g25430)),AT1G71390),(BnaN07g25420,(BnaN04g15330,BnaN02g19970)),Bra003927);
