# Supplementary material for: Genomic evidence for genes encoding leucine-rich repeat receptors linked to resistance against the eukaryotic extra- and intracellular Brassica napus pathogens Leptosphaeria maculans and Plasmodiophora brassicae
Source: PLoS One. 2018 Jun 1;13(6):e0198201. doi: 10.1371/journal.pone.0198201 (PMC5983482; doi:10.1371/journal.pone.0198201)
Supplement: S2 Appendix — The package GenomicRanges was used to compare genomic intervals of genes and resistance loci. Results were subsequently automatically inserted into pre-existing tables. (ZIP) [file pone.0198201.s010.zip › S2Appenix-Code_090517.docx]

**S2 Appendix.** **R code to generate hit tables based on published mapping information.** The package GenomicRanges was used to compare genomic intervals of genes and resistance loci. Results were subsequently automatically inserted into pre-existing tables.

# Set working directory

setwd("~/Papers/RLPaper/GenomicRanges")

# Install GenomicRanges once

source("http://bioconductor.org/biocLite.R")

biocLite("IRanges")

biocLite("GenomicRanges")

biocLite("rtracklayer")

# Execute package every time

library(GenomicRanges)

# Load data files into R; TraitQTL data relate to Table S3 and GeneList to TableS1

TraitQTL <- read.table("ClubrootQTL.csv",sep=",",header=T)

Genelist <- read.table("NLRoutputRearranged3.csv",sep=",",header=T)

# Generate genomic intervals using the GenomicRanges package and check conversion using the head() function

TraitQTLrd <- GRanges(TraitQTL)

# head(TraitQTLrd)

GENEgr <- GRanges(Genelist)

# head(GENEgr)

# Compare query and subjects

hits <- findOverlaps (TraitQTLrd, GENEgr, ignore.strand=T)

# head (hits)

# hits

write.table(hits, "ClubrootNLRdata.txt",sep=",")

## Contingency test

DiseaseQTL <- read.table("ClubrootStats3.csv",sep=",",header=T)

# DiseaseQTL

results <- chisq.test(DiseaseQTL)

# results

results$expected

# Read in results table

x <- read.table("ClubrootNLRdata.txt",sep=",",header=T)

# head(x)

# Find unique query hits

y <- unique(x[,1])

y

a <- unique(x[,2])

a

length(a)

# Generate list

z <- list(length(y))

# head(z)

# Define a list of all observations

for(i in 1:length(y)) z[[i]] <- x[as.vector(which(x[,1]==y[i])),2]

res <- as.data.frame(cbind(y,z))

# head(res)

# class(res$z)

# Read in table with gene-IDs

b <- read.csv("NLRoutRearranged.csv")

# head(b)

# Find gene-IDs by applying row numbers of x[,2] to b

f <- z

# head(f)

for(i in 1:length(f)) f[[i]] <- paste(as.character(b[z[[i]],8]), collapse=", ")

g <- as.data.frame(cbind(y,unlist(f)))

colnames(g) <- c("QTLhit","geneID")

# head(g)

write.csv(g, "ClubrootNLRgeneIDs.csv")

# Incorporate geneIDs (g$V2) into LarkanQTLtable

h <- read.csv("ClubrootTableRLPsp.csv")

# head(h)

j <- cbind(h,NLR=NA)

# head(j)

k <- j

# Populate geneIDs into table

# class(g$QTLhit)

k[as.numeric(as.character(g$QTLhit)),7] <- as.character(g$geneID)

# head(k)

write.csv(k, "ClubrootFinalTable.csv")
